# Cholera in Cincinnati

**Published:** 1850-10

**Authors:** 


*Cholera in Cincinnati.*—The Western Lancet states that the cholera has entirely disappeared from Cincinnati. Dysentery and diarrhœa prevail, but not to a greater extent than frequently is the case at this season. The deaths from cholera during the months of June, July, and August, amount to *fourteen hundred*, as certified by the Board of Health.

